# Opportunity for Collaboration: A Conceptual Model of Success in Tobacco Control and Cancer Prevention

**Published:** 2011-12-15

**Authors:** Frances A. Stillman, Carol L. Schmitt, Scott R. Rosas

**Affiliations:** The Johns Hopkins Bloomberg School of Public Health; RTI International, Washington, DC; Concept Systems, Inc, Ithaca, New York

## Abstract

**Introduction:**

Collaborations between cancer prevention and tobacco control programs can leverage scarce resources to address noncommunicable diseases globally, but barriers to cooperation and actual collaboration are substantial. To foster collaboration between cancer prevention and tobacco control programs, the Global Health Partnership conducted research to identify similarities and differences in how the 2 programs viewed program success.

**Methods:**

Using concept mapping, cancer prevention and tobacco control experts generated statements describing the components of a successful cancer prevention or tobacco control program and 33 participants sorted and rated the final 99 statements. Multidimensional scaling analysis with a 2-dimensional solution was used to identify an 8-cluster conceptual map of program success. We calculated Pearson correlation coefficients for all 99 statements to compare the item-level ratings of both groups and used* t* tests to compare the mean importance of ratings assigned to each cluster.

**Results:**

Eight major clusters of success were identified: 1) advocacy and persuasion, 2) building sustainability, 3) partnerships, 4) readiness and support, 5) program management fundamentals, 6) monitoring and evaluation, 7) utilization of evidence, and 8) implementation. We found no significant difference between the maps created by the 2 groups and only 1 mean difference for the importance ratings for 1 of the clusters: cancer prevention experts rated partnerships as more important to program success than did tobacco control experts.

**Conclusions:**

Our findings are consistent with those of research documenting the necessary components of successful programs and the similarities between cancer prevention and tobacco control. Both programs value the same strategies to address a common risk factor: tobacco use. Identifying common ground between these 2 research and practice communities can benefit future collaborations at the local, state, tribal, national, and international levels, and inform the broader discussion on resource sharing among other organizations whose mission focuses on noncommunicable diseases.

## Introduction

Noncommunicable diseases (NCDs) are the leading cause of illness and death in high-income countries and are emerging as a leading cause of death in low- to middle-income countries (1). However, countries at all income levels (high, middle, and low) often lack sufficient resources and capacity to address this issue (2-4).

There have been notable examples of collaboration between cancer prevention and tobacco control organizations in the United States and Canada (5). Tobacco control is the highest priority prevention component to reduce cancer mortality and a major modifiable risk factor to control the growing epidemic of NCDs (6). A focus on common modifiable risk factors is an opportunity to maximize available resources for NCDs and can serve as a unifying framework for collaborative organizational relationships that can increase the efficiency, effectiveness, and sustainability of public health efforts (7-9). Although the need to collaborate is well recognized, substantial barriers to collaborations exist between public health organizations, including tobacco and cancer control programs. By focusing on the similarities between cancer prevention and tobacco control, much can be learned about future efforts for these programs and for the NCD agenda.

Despite their common focus on reducing tobacco use, substantial misunderstandings and myths have created barriers to working relationships between the tobacco control and the cancer prevention communities (10). Ameliorating these barriers requires sharing information, building trust, understanding differing organizational cultures and processes, and emphasizing the fundamental interconnections of these 2 disciplines (10). Working toward a shared vision of how resources are appropriated is also essential because competition for scarce resources is an important barrier to collaboration (11) and without systematic planning, programs often prioritize treatment-oriented interventions at the expense of primary prevention (including tobacco control), early detection, and palliative care (6). Furthermore, differing expectations for success, as defined by a treatment-oriented versus a prevention-oriented program, are difficult to reconcile. For example, a cancer treatment with a 10% success rate is considered poor, whereas a population-wide intervention such as a tax increase that results in a 5% or 10% reduction in tobacco use is a major success (11). Moreover, an effective treatment quickly yields reductions in cancer deaths, whereas an effective tobacco prevention intervention does not yield a reduction in cancer incidence and illness for decades. A shared definition of program success could provide a framework for collaboration between cancer prevention and tobacco control programs.

Our objective was to define a successful program in terms of how experts in cancer prevention and tobacco control described, categorized, and rated its components. We used concept mapping to examine the extent to which these 2 fields shared a common conceptual framework for program success.

## Methods

We used concept mapping to identify and organize best practices common to both the tobacco control and cancer prevention communities. Concept mapping is a type of Delphi method, a participatory process in which findings represent the ideas of those who contribute. As a mixed-methods planning and evaluation approach, it integrates familiar qualitative group processes (brainstorming, categorizing ideas, and assigning value ratings) with multivariate statistical analyses to help a group describe its ideas on any topic of interest and represent these ideas visually through a series of maps (12,13).

### Participants and procedures

During May to June 2008, a convenience sample of 70 cancer prevention and tobacco control program grantees and stakeholders associated with the Pfizer Global Health Partnerships (GHP) initiative, which provided 3 years of funding to 31 diverse tobacco control and cancer prevention interventions in 46 countries, were contacted by e-mail and asked to participate in a study about their perceptions of a successful tobacco or cancer prevention program. The e-mail included the address of a project-specific website where participants were asked to complete the following prompt: "Given your expertise and/or knowledge of the Global Health Partnerships program, a specific characteristic of a successful tobacco and/or cancer prevention program would be . . .". This process yielded 161 statements, which were reduced to 99 statements once duplicates were eliminated.

The original 70 participants were subsequently contacted by e-mail and asked to rate each statement on a scale from 1 (relatively unimportant) to 5 (very important) and to sort the entire list of ideas into groups or themes based on similarity of ideas. The rating and sorting tasks required more time and effort to complete than the brainstorming task, and we anticipated attrition among the original participants. As a result, Pfizer GHP program stakeholders nominated an additional 10 leaders in tobacco and cancer control, who were also invited to participate. Participants identified their field of expertise, the geographic area in which they worked, and whether they were GHP grantees.

### Analyses

We used the Concept System (Concept Systems, Inc, Ithaca, New York) to facilitate the concept-mapping process. This software uses multidimensional scaling and hierarchical cluster analysis to integrate the sorting information from each participant and develop a series of easily readable concept maps and reports. The maps provide a visual representation of how participants sorted and rated the statements generated during the brainstorming phase. For these analyses we generated 3 maps: 1 using all respondent data, 1 using data from 19 tobacco control participants, and 1 using data from 12 cancer prevention participants.

The analysis aggregated the sort information from each participant to construct a 99 x 99 matrix of similarities; values in the cells represented the extent to which individuals within the group sorted the 2 ideas together. The total similarity matrix was analyzed by using nonmetric multidimensional scaling (MDS) analysis with a 2-dimensional solution. The 2-dimensional solution yields a configuration in which statements grouped together most often are located more closely in 2-dimensional space than those grouped together less frequently. The 2-dimensional (x,y) configuration resulting from the MDS analysis was the input for the hierarchical cluster analysis. To determine the best-fitting cluster solution, we examined a range of possible cluster solutions suggested by the analysis. We took into account the fit of the contents within clusters and an overall understanding of public health program design and evaluation. Detailed descriptions of the procedures and analyses used in concept mapping are described elsewhere (12,13). Although better fit and greater similarity between the input data and output representation might be observed using more than 2 dimensions, the current approach is generally regarded as appropriate for generating the most parsimonious and interpretable results for concept mapping. Trochim (12) found the 2-dimensional plot of brainstormed statements to be acceptable, especially when the MDS configuration is subjected to cluster analysis.

To further examine the similarity or agreement in the sorted arrangements produced by tobacco control and cancer prevention participants, we compared the aggregated sort matrices of the 2 groups. Each group's symmetric matrix was arranged in a vector and a Pearson product moment correlation between the pairs of values in the 2 columns was computed as a measure of congruence of the interrelationships among statements. We also calculated a Pearson correlation for all 99 statements to assess the overall correspondence of the item-level ratings between both groups. Finally, we computed independent sample *t* tests to compare the mean importance ratings for each cluster to determine any differences between cluster ratings of tobacco control and cancer prevention experts.

GHP stakeholders and evaluators reviewed the maps and developed a preliminary interpretation. The final map labels and interpretations were completed after the maps were presented and additional comments solicited from GHP stakeholders and grantees at an August 2008 evaluation workshop.

## Results

**Box 1.** Participant Expertise, Experience, and Geographic Location (N = 33)

**Area of Expertise**	**Years' Experience**	**Geographic Location**
Cancer: 12 (36%) Tobacco: 19 (58%) NR: 2 (6%)	≤5: 8 (24%) >5: 20 (61%) NR: 5 (15%)	North America: 10 (30%) Asia: 6 (18%) Europe: 6 (18%) Latin America: 3 (9%) North Africa: 1 (3%) Multiple areas: 4 (12%) NR: 3 (9%)
Abbreviation: NR, participant did not respond to this question.		

Thirty-seven of the 70 original invited participants (53%) completed the brainstorming task. Of the 80 participants (the 70 original participants plus an additional 10) invited to complete the sorting and rating task, 33 (41%) completed it, a response rate comparable to published web-based survey response rates of 31% (13). Approximately 50% of the participants who completed the sorting and rating task were recipients of GHP funding. Their areas of expertise, experience, and geographic areas are described in Box 1.

The final map that resulted from the sorting and rating task (Figure) shows that 8 major clusters (or constructs) were considered essential in defining what constitutes a successful tobacco control or cancer prevention program. Cluster location also reflects relationship between these clusters to show how these clusters are connected to each other. Three sectors structure the 8 clusters into a 3-section map: the human and relational sector, program capacity sector, and technical and scientific sector.

**Figure.**
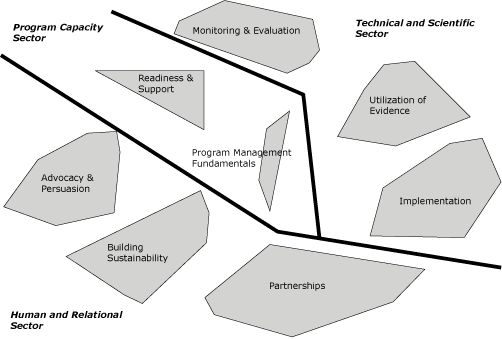
Overall concept map of successful program components.

Moving from the lower left side of the map through each sector, the following summarizes the 8 clusters:

### Human and relational sector

**Advocacy and persuasion**

The statements in this cluster focused on understanding context (eg, the political environment) and using information about context to increase program effectiveness and sustainability. Themes raised included using program outcomes to advocate for interventions, policy change, and increased funding. Having organizational management with strategic and visionary leadership capabilities was found to be an important component of this cluster.

**Building sustainability**

The statements in this cluster described what participants viewed as the most important factors to program sustainability. Many of these statements incorporated concepts from the other 7 clusters. Developing future leadership was a key theme and included recognition that sustainability requires leadership among multiple organizations and within affected populations. Institutional capacity and skills, including the development of systems to ensure that staff skills are sustained and emerging knowledge is incorporated into program practice, were also considered important components of sustainability building.

**Partnerships**

The statements in this cluster focused on building networks for program development, implementation, and dissemination of findings. The partnerships identified include the target population, allied organizations, government and civil society, and technology networks. Several statements cited the benefits of partnerships including knowledge flow, coordination, and the potential to reduce duplication of efforts.

### Program capacity sector

**Readiness and support**

The statements in this cluster focused primarily on the need to assess the resources that a program has available and can potentially access, including staff skills, knowledge and training, and technical assistance.

**Program management fundamentals**

The statements in this cluster were few and relatively diverse. However, the major themes focused on the importance of not only having sufficient resources but also being able to access and manage those resources, including money, staff, and equipment.

### Technical and scientific sector

**Monitoring and evaluation**

The statements within this cluster focused on evaluation components (eg, indicators), practices (incorporating evaluation findings into practice; ensuring that an evaluation is objective and is adequately funded; using technology), and specific indicators (policy change).

**Use of evidence**

This cluster was located close to the monitoring and evaluation cluster, meaning that participants saw them as similar. The statements focused on the importance of using evidence from research, practice, and evaluation to guide program design and implementation.

**Implementation**

The statements in this cluster described what participants considered key guiding principles for program implementation. These principles emphasized the use of a policy-based approach; identifying and tailoring the program to the population served; and specific operational guidelines, such as identification and education of staff with appropriate skills and the inclusion of conflict-of-interest policies.

**Box 2.** Top-Ranked Characteristics of a Successful Tobacco or Cancer Prevention Program, by Cluster Within Sector


**Human and relational sector**

**Advocacy and persuasion cluster** Sharing outcomes (both intended and unintended). Using results to advocate for the program. Using results to influence future legislation. **Building sustainability cluster** Having strong and effective collaborative leadership. Focusing on changes at the policy level. Creating new policy-oriented champions, organizations, and coalitions that will make long-term contributions to these fields. **Partnerships cluster** Enabling stakeholders to develop a shared understanding of what must be done and how best to do it. Being inclusive of the target audience. Involving a collaborative process with multiple sectors, organizations, and stakeholders in all phases.

**Program capacity sector**

**Readiness and support cluster** Building on knowledge of what is already being done in the field. Identifying the existing resources that can be used and built on. Having good analytic capacity to identify new opportunities. **Program management fundamentals cluster** Having staff with sufficient management skills to conduct their program. Understanding the health care system of the country or region you are working with. Translating evidence-based interventions into policy and program.

**Technical and scientific sector**

**Monitoring and evaluation cluster** Having clear and measurable goals and objectives. Having the capacity to monitor and evaluate program components. Including both process and impact/outcome evaluation. **Utilization of evidence cluster** Ensuring the program is of the highest quality possible. Addressing a clearly articulated need. Using evidence to guide the operation of the program. **Implementation cluster** Having staff with the technical skills needed to conduct a program. Promoting adoption of proven cancer prevention and tobacco control policies. Using time and resources efficiently for planning, implementation, and program sustainability.

The 3 highest-rated statements within each cluster are listed in Box 2. A significant relationship was found between how tobacco control and cancer prevention experts structured the 99 items (r_4948_ = .73, *P* < .001) and how important they rated them (r_29_ = .65, *P* < .001) on the 1 to 5 scale. At the cluster level, where item ratings were aggregated within the cluster to which they were assigned, 1 significant difference was observed: cancer prevention experts rated the importance of partnerships significantly higher (mean, 4.12) than tobacco control experts did (mean, 3.94; t_34_ = 2.20; *P* < .05). Although the difference in absolute values appears small, the cluster means are derived from the average across both the participants and items within a cluster. This produces a very narrow range of means across the clusters, so even slight differences in averages between clusters can be meaningful (16).

## Discussion

In this analysis, cancer prevention and tobacco control experts identified a set of common components that characterized successful programs for both fields. Rather than focusing on the standard outcomes most frequently considered to be evidence of success, such as decreases in prevalence, incidence, illness, and death, participants focused primarily on the resources and capacity a program needs to effectively plan, implement, and sustain program activities and on the use of evidence-based information to guide program activities and achieve program goals. Since "collaboration takes a significant investment of time to build trust, to overcome differences in perspective, and to develop a workable, concrete agenda for joint action" (12), the time to build collaboration is during the initial planning phase of program development. This process could help reduce the cost of building separate resources and capacity for cancer prevention and tobacco control programs.

Both cancer prevention and tobacco control experts perceived all 8 clusters as important to success, rating all clusters between 4.0 and 4.2. The only significant difference in ratings focused on partnerships. The lack of difference was surprising because cancer prevention programs traditionally focus on program implementation such as screening and treatment, whereas tobacco control programs have historically focused on policy change (12). It does, however, suggest opportunities for each field to learn from the other. For example, cancer control programs seeking to integrate best practices for cancer screening, treatment, and palliative care into systematic practice could adapt some of the strategies tobacco control has successfully used toward smoke-free air policies and adoption of cessation practices in health care settings. Likewise, tobacco control programs that could be rejected for political and policy reasons may be more acceptable to national governments if housed within a comprehensive cancer prevention program (7).

Similarly, both sets of experts acknowledged the importance of surveillance and monitoring, including the importance of measuring both process and outcome indicators. The focus on leadership within both the *sustainability* and *advocacy and persuasion* clusters presents an opportunity to develop and collect common process indicators. The higher perceived importance that cancer experts assigned to partnerships may indicate how essential collaborations are to effectively integrate the diverse approaches and organizations required to successfully implement all 4 components of cancer control — from prevention to early detection through treatment and palliative care — and further to integrate multiple and widely varied cancer types within a comprehensive cancer model. Cancer experts can use their expertise and experience in partnership building to facilitate collaborations with tobacco control programs.

Our results represent the views and opinions of a small sample of experts in tobacco control and cancer prevention. However, concept mapping is a technique that has been used to develop frameworks that can be adapted to similar contexts and thus does provide a basis for generalization. In this case, we are comparing tobacco and cancer organizations. The results can be generalized to other groups or organizations that are working on prevention and control issues, especially those related to NCDs (http://www.socialresearchmethods.net/kb/external.php3) and thus could prove useful as the dialogue concerning NCDs continues to develop.

Most low- and middle-income countries lack the basic knowledge and capacity to plan and implement separate cancer prevention and tobacco control programs (6) and thus also lack the resources, capacity, and political will to implement comprehensive programs to address modifiable NCD risk factors (16). Organizations should plan integrated interventions and pool limited resources before separate programs are institutionalized. Collaborative relationships also engage stakeholders with access to global and national leadership. Building collaborations during the planning phase is consistent with World Health Organization (WHO) recommendations for national cancer prevention program planning in countries of all income levels. Those recommendations include building the capacity to implement and sustain core functions of a comprehensive cancer control program that includes tobacco control as a key component of prevention (6). Collaboration building is also consistent with WHO recommendations for preventing NCDs through an integrated approach to addressing modifiable risk factors (17).

Tackling modifiable risk factors through prevention is essential to successfully ameliorating the coming epidemic of NCDs in low- and middle-income nations (17). However, collaborative relationships between allied organizations are essential to effective use of limited resources; without comprehensive planning, treatment may be prioritized at the expense of prevention (6). Our findings demonstrate that cancer prevention and tobacco control programs have the opportunity to integrate their approaches to cancer and more broadly to NCD prevention as low- and middle-income countries develop comprehensive plans to address the modifiable risk factor they share: tobacco use.
